# Peace

**Published:** 1889-10-05

**Authors:** 


### Words of Consolation.

### PEACE.

The word "peace" brings to the mind of busy, tired people the desire for a blessing which they all long to enjoy, as they hurry to and fro with restless feet, with care and anxiety of heart, and a ceaseless striving for daily bread. Even the noise and excitement of pleasure-seeking only carries them farther from their goal; all such distractions are enemies to true peace of mind. Indeed the world never pretends to give such a boon to the weary. It may speak or write about a tranquil life which at the best is only a state free from outward trials and disturbances, but the word peace as a promise is never mentioned; it is not in its vocabulary, therefore it cannot offer what it does not possess.

On the other hand, the Christian enjoys a peaceful life amidst outward disquietude. He, like other men, has his daily task of work to do, his constant employments are often fatiguing and harrassing, full of interruptions, and sometimes distasteful in themselves. Yet, through and under all, there is inward joy and peace. He has given himself into God's care, he trusts in Him, and at all times rests in the gracious promise, "Thou wilt keep him in perfect peace whose mind is stayed on Thee: because he trusteth in Thee." He has gained everlasting strength from the Lord Jehovah to carry him through the heavy storms of affliction, such as grievous sickness or the loss of those he dearly loves. For the Christian is no Stoic: he weeps as other men weep at the parting grave, but sorrows not as those without hope: he knows that God Himself directs the blow which cripples him either in body or in heart, and though he may never thoroughly recover from the effects in this life yet he is sure that the Heavenly Architect who polishes "with many a blow and biting sculpture" the living stones with which the Heavenly Salem is built, is only preparing him for a place among the number of those who are "made perfect through suffering."

And now let us see how this inward peace bears fruit in the outward life of the true believer. He takes with cheerfulness whatever falls to his lot, his temper is even and serene, little irritations pass by without stinging him, labour becomes light, and his demeanour is respectful, kind, and courteous to all. He adorns the doctrine which he professes, and is a bright example to the newly awakened, who find encouragement in the calm contentment of his face. But his internal peace and rest is far greater than any onlooker can guess: it is the "peace of God which passeth all understanding," a gift as wonderful as the giver. This it is which amidst all trials and distresses, all troubles and doubts, is as the shadow of a great rock in a thirsty land, cooling, protecting, invigorating who flee to it for shelter.

There are, in this loud stunning tide
Of human care and crime,
With whom the melodies abide
Of th' everlasting chime;
Who carry music in their heart
Through dusky lane and wrangling mart,
Plying their daily task with busier feet,
Because their secret souls a holy strain repeat.

